# Self-Management Using eHealth Technologies for Liver Transplant Recipients: Scoping Review

**DOI:** 10.2196/56664

**Published:** 2024-07-04

**Authors:** Soo Hyun Kim, Kyoung-A Kim, Sang Hui Chu, Hyunji Kim, Dong Jin Joo, Jae Geun Lee, JiYeon Choi

**Affiliations:** 1 School of Nursing, Johns Hopkins University Baltimore, MD United States; 2 College of Nursing, Suwon Women's University Suwon Republic of Korea; 3 College of Nursing, Mo-Im Kim Nursing Research Institute, Yonsei University Seoul Republic of Korea; 4 School of Nursing, Yale University Orange, CT United States; 5 Department of Surgery, College of Medicine, Yonsei University Seoul Republic of Korea; 6 College of Nursing, Mo-Im Kim Nursing Research Institute, Institute for Innovation in Digital Healthcare, Yonsei University Seoul Republic of Korea

**Keywords:** liver transplantation, self-management, transplant management, eHealth, eHealth technology, digital health, review, mobile phone, liver transplant, liver disease, scoping review

## Abstract

**Background:**

Liver transplantation has become increasingly common as a last-resort treatment for end-stage liver diseases and liver cancer, with continually improving success rates and long-term survival rates. Nevertheless, liver transplant recipients face lifelong challenges in self-management, including immunosuppressant therapy, lifestyle adjustments, and navigating complex health care systems. eHealth technologies hold the potential to aid and optimize self-management outcomes, but their adoption has been slow in this population due to the complexity of post–liver transplant management.

**Objective:**

This study aims to examine the use of eHealth technologies in supporting self-management for liver transplant recipients and identify their benefits and challenges to suggest areas for further research.

**Methods:**

Following the Arksey and O’Malley methodology for scoping reviews, we conducted a systematic search of 5 electronic databases: PubMed, CINAHL, Embase, PsycINFO, and Web of Science. We included studies that (1) examined or implemented eHealth-based self-management, (2) included liver transplant recipients aged ≥18 years, and (3) were published in a peer-reviewed journal. We excluded studies that (1) were case reports, conference abstracts, editorials, or letters; (2) did not focus on the posttransplantation phase; (3) did not focus on self-management; and (4) did not incorporate the concept of eHealth or used technology solely for data collection. The quality of the selected eHealth interventions was evaluated using (1) the Template for Intervention Description and Replication guidelines and checklist and (2) the 5 core self-management skills identified by Lorig and Holman.

**Results:**

Of 1461 articles, 15 (1.03%) studies were included in the final analysis. Our findings indicate that eHealth-based self-management strategies for adult liver transplant recipients primarily address lifestyle management, medication adherence, and remote monitoring, highlighting a notable gap in alcohol relapse interventions. The studies used diverse technologies, including mobile apps, videoconferencing, and telehealth platforms, but showed limited integration of decision-making or resource use skills essential for comprehensive self-management. The reviewed studies highlighted the potential of eHealth in enhancing individualized health care, but only a few included collaborative features such as 2-way communication or tailored goal setting. While adherence and feasibility were generally high in many interventions, their effectiveness varied due to diverse methodologies and outcome measures.

**Conclusions:**

This scoping review maps the current literature on eHealth-based self-management support for liver transplant recipients, assessing its potential and challenges. Future studies should focus on developing predictive models and personalized eHealth interventions rooted in patient-generated data, incorporating digital human-to-human interactions to effectively address the complex needs of liver transplant recipients. This review emphasizes the need for future eHealth self-management research to address the digital divide, especially with the aging liver transplant recipient population, and ensure more inclusive studies across diverse ethnicities and regions.

## Introduction

### Background

As the last treatment resort for individuals with end-stage liver diseases or liver cancer [1], liver transplantation (LT) has become one of the fastest-growing solid organ transplant procedures worldwide. Since its first case in 1963, LT has evolved into a more viable treatment option for those living with end-stage liver conditions. In the United States, >9000 individuals receive LTs annually [2]. In South Korea, the number of LT cases increased from 1066 in 2010 to 1515 in 2021 [3]. Over the past decades, there has been notable progress in the success of LT surgery and long-term survival rates [4]. In the United States, the 1-year survival rate reached 89%, and the 5-year survival rate is >74%, although variations exist depending on donor types, underlying diagnoses, recipient age, and region [5].

Despite the improving trend of posttransplant survival, optimizing the benefits of LT remains a complex and challenging issue for LT recipients. Research on post-LT outcomes to date has primarily focused on graft function and overall survival, corresponding to rapidly advancing surgical techniques and drug development [6]. Relatively little attention has been paid to promoting posttransplant self-management and its impact on long-term quality of life (QOL) [7]. After transplant, LT recipients must manage risks of complications, such as intestinal adhesion, bleeding, and bile leakages, and maintain a balance between graft failure risks and the side effects of immunosuppressant therapy, which often necessitate frequent dosage changes [8,9]. Additional lifelong challenges include management of common side effects of immunosuppressant therapy, such as hyperlipidemia, high blood pressure, chronic kidney failure, obesity, diabetes, and infection [10,11].

To address these challenges and maximize the benefits of LT, vigilant posttransplant self-management is crucial. Self-management has been defined in the literature as a comprehensive process that encompasses focusing on one’s illness needs (eg, acquiring knowledge and skills, monitoring symptoms, problem-solving, and decision-making), using available resources, building partnerships with health care providers (HCPs), and integrating illness management into daily life [12-14]. For LT recipients, major self-management issues include symptom monitoring, medication management, and engaging in healthy lifestyles after transplant [15-17]. Furthermore, LT recipients must navigate complex health care systems, face changes in social roles, and cope with uncertainty and mental distress associated with the ever-present risk of graft rejection [18]. During the COVID-19 pandemic, with reduced human contact support and the strained health care systems reallocating resources, self-management became more challenging for immunocompromised individuals such as LT recipients [19]. However, the increased accessibility to the internet and digital devices, coupled with the challenges posed by the pandemic, has rapidly escalated interest in interventions using the internet to promote or manage health (eHealth). These technologies hold the potential to overcome challenges related to resource allocation, geographical accessibility, and health care cost.

### Prior Work

eHealth, defined as the use of the internet and communication technologies to deliver and improve health care services [20], has been rapidly expanding to promote self-management in acute and chronic conditions [21-23]. However, the application of eHealth for LT recipients has been relatively slow due to the complexity of LT management, the need for close physical examinations, and the importance of building rapport with HCPs for lifelong posttransplant care [24]. Although there have been studies investigating the application of eHealth among solid organ transplant recipients [25,26], to date, no review has specifically focused on eHealth for self-management support in LT recipients. Therefore, this study aimed to map the current state of the literature on self-management using eHealth technologies for LT recipients and assess their benefits and challenges to suggest areas needing further investigation in the field.

### Goal of This Study

This review aimed to examine the current literature on eHealth-based self-management among adult LT recipients and its associated factors by addressing the following questions: (1) what are the characteristics and associated factors of eHealth strategies in the adult LT recipient population? (2) how effective and feasible are eHealth-based self-management interventions after LT? and (3) what are the future potential and challenges of eHealth in facilitating self-management among this population? By mapping the existing literature through this scoping review, we aimed to identify gaps and propose future directions for the development and application of eHealth-based self-management interventions for adult LT recipients.

## Methods

### Study Design

We conducted a scoping review based on the 6-stage scoping review framework by Arksey and O’Malley [27]. This methodology was chosen to examine the breadth and depth of knowledge in an emerging field of research. We used the population, concept, and context criteria to devise the research question for this review: adult LT recipients (population), eHealth (concept), and facilitating self-management (context) [28]. Given that previous studies on self-management among LT recipients have primarily concentrated on medication adherence, alcohol recidivism, and healthy lifestyle maintenance [15], our review specifically focused on these areas of self-management.

### Databases and Search Terms

The search and screening procedure of this scoping review adhered to the guidelines provided by the PRISMA (Preferred Reporting Items for Systematic Reviews and Meta-Analyses) checklist. We systematically searched 5 electronic databases: PubMed, CINAHL, Embase, PsycINFO, and Web of Science. The search terms comprised a combination of the following: *adult LTR*, *e-Health*, and *self-management*. Our concept of eHealth encompasses a range of technology, including telehealth and internet-, computer-, or mobile-based health, and strategies using video, audio, SMS text messaging, wearables, and virtual reality. To conduct the search on self-management, we included and modified terms such as *alcohol*, *nutrition*, *exercise*, *physical activity*, *medication*, *medication adherence*, *self-care*, *self-management*, and *health behavior*. Our search spanned inception to June 19, 2023, without any restrictions on specific study design. We validated our search strategies through consultation with a university librarian and present the detailed search strategies in Multimedia Appendix 1.

### Inclusion and Exclusion Criteria

Articles were included if they were studies that (1) examined or implemented eHealth-based self-management, (2) included LT recipients aged ≥18 years, and (3) were published in a peer-reviewed journal. We excluded studies that (1) were case reports, conference abstracts, editorials, or letters; (2) did not focus on the posttransplantation phase; (3) did not focus on self-management; and (4) did not incorporate the concept of eHealth or solely used technology for the purpose of data collection. We also considered studies that targeted various solid organ transplant recipients provided they included adult LT recipients. Our review specifically targeted adult patients in the post-LT phase as LT in children often involves dissimilar underlying conditions for transplant, such as biliary atresia [29], and post-LT self-management in pediatric patients may present additional challenges related to life stage development.

### Article Selection

**Figure 1:**
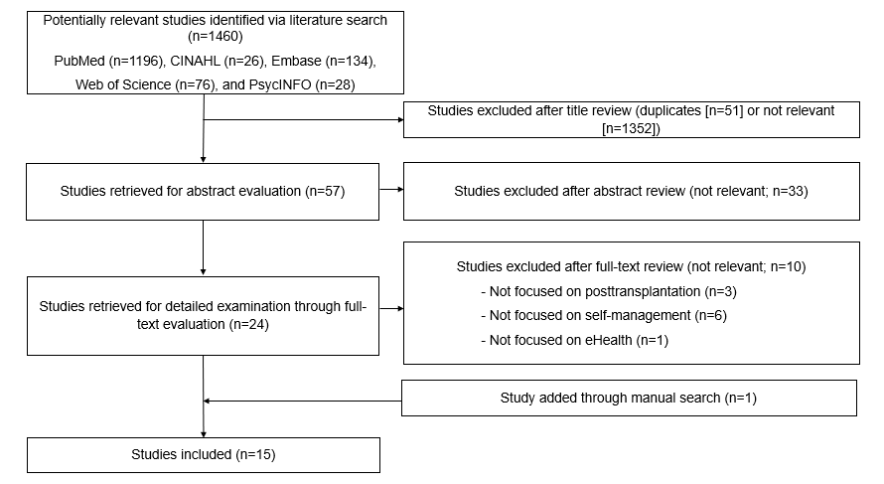
PRISMA (Preferred Reporting Items for Systematic Reviews and Meta-Analyses) flowchart.

A total of 1460 articles were identified. After removing duplicates (51/1460, 3.49%), 1409 articles were imported to Microsoft Excel (Microsoft Corp) for screening using the inclusion and exclusion criteria. First, 2 authors (SHK and HK) independently screened the titles and excluded 95.95% (1352/1409) of the articles, which did not include LT recipients or were not about eHealth or self-management (eg, drug trials and surgery). This yielded 57 articles. In the second stage, 2 authors independently reviewed the abstracts of the 57 articles. Finally, a full-text review was conducted on 42% (24/57) of the articles. The references of the selected articles were manually examined to search for additional articles that could be eligible for this review. Consequently, 1 article was added through the manual search. A total of 15 articles were included in the final sample. The whole process was supervised by the corresponding author, and disagreements between authors were discussed in research team meetings until a consensus was reached. Figure 1 illustrates the PRISMA flow diagram.

### Data Analysis

Due to the heterogeneity of the interventions and measured outcomes, a meta-analysis or meta-synthesis of the results was not feasible. Instead, we presented the organized data in tables, which include the summaries of the descriptive and intervention studies and a detailed summarization of the interventions.

The following information was extracted using data-charting forms: first author’s last name, publication year, country, study design, sample characteristics (sample size, average age, percentage of male individuals, and time since transplant), type of technology, variables with measures, and outcomes. For intervention studies, we analyzed the details of the intervention and control groups and extracted the following characteristics of the interventions: duration, providers, adherence, adverse events, and reasons for attrition.

We also evaluated the quality of the interventions reported in these studies using the Template for Intervention Description and Replication (TIDieR) checklist and guide [30]. The TIDieR checklist and guide address the items of interventions that need to be thoroughly described to enhance the replicability of trials and facilitate the appraisal of intervention reporting. To gauge the comprehensiveness of intervention content, we analyzed whether the interventions addressed the 5 core self-management skills identified by Lorig and Holman [14]. These core skills, which include problem-solving, decision-making, using resources, partnering with HCPs, and taking action, have been considered as fundamental elements of self-management in the literature that examines the definitions, components, and processes of self-management [12,13,31,32].

### Ethical Considerations

No ethics approval was required for this scoping review as published articles rather than primary data were used in the analysis.

## Results

### Study Characteristics

The general characteristics of the 5 descriptive studies and 10 intervention studies are summarized in Tables 1-4. Of the 5 descriptive studies, 2 (40%) were qualitative and 3 (60%) were quantitative studies. Of the 10 intervention studies, 4 (40%) were randomized controlled trials (RCTs), 4 (40%) were prospective observational studies with cohorts, 1 (10%) was a study with a historical control group, and 1 (10%) was a single-group qualitative evaluation of a feasibility study. A total of 67% (10/15) of the studies were published in 2020 or later, with all studies (15/15, 100%) being published after 2016. Most studies were conducted in North America (7/15, 47% in the United States and 1/15, 7% in Canada) and Europe (1/15, 7% in Austria; 2/15, 13% in Belgium; and 1/15, 7% in Spain). The sample sizes of the selected studies varied, ranging from 19 to 710 participants, with the average age of the participants ranging from 46 to 63 years.

**Table 1:** Characteristics of the descriptive studies.

Study	Year	Country	Study design	Sample size	Age (y)	Sex (male; %)	Time since transplant
Lieber et al [33]	2021	United States	Qualitative study	20	Median 61	65	Not reported
Maroney et al [34]	2021	United States	Quantitative survey	178 KTRs^a^ and 110 LTRs^b^	Mean 52.6	54.5	46.88% between 1 and 2 years after transplant
Mathur et al [35]	2021	Canada	Qualitative study	5 LTRs out of 21 SOTs^c^	Mean 47	48	2 years to 8 years
Vanhoof et al [36]	2018	Belgium	Cross-sectional descriptive study	30 LTRs out of 122 SOTs	Mean 55.9	57.4	Median 6 years
Wedd et al [37]	2019	United States	Cross-sectional descriptive study	455 KTRs and 255 LTRs	Median 49 (KTRs) and 53 (LTRs)	55.2 (KTRs) and 59.6 (LTRs)	6 months before transplant to 2 years after transplant (study period)

^a^KTR: kidney transplant recipient.

^b^LTR: liver transplant recipient.

^c^SOT: solid organ transplant.

**Table 2:** Results of the descriptive studies.

Study	Type of technology	Variable (measure)	Outcome
Lieber et al [33]	App	Smartphone use Acceptability and preference regarding smartphone app: thematic analysis of semistructured interviews	90% of participants used a smartphone. The most frequently cited purpose of smartphone use for posttransplant recovery was setting alarm reminders to take medications. 80% of participants were interested in using an app to connect with peer LT^a^ recipients and HCPs^b^, gain educational information and support medication taking, and log biometric data.
Maroney et al [34]	Patient portal (internet or smartphone)	eHealth literacy: eHEALS^c^ Routine patient portal use (defined as routine if use frequency was every day, weekly, or monthly)	eHealth literacy: KTRs^d^>LTRs^e^ (*P*=.04) eHealth literacy: college education or higher>high school education or lower (*P*<.001) eHealth literacy was higher in those with mobile internet access than in those without (*P*=.04) Routine users had higher eHealth literacy than nonroutine users and nonusers (eHEALS score 31.97 vs 29.97 vs 28.20; *P*<.001).
Mathur et al [35]	Digital health tools	Barriers and motivators to PA^f^ Core features of a digital health tool to support PA: thematic analysis of semistructured interviews	Barriers to PA included risk aversion, managing nonlinear health trajectories, physical limitations, and lack of access to appropriate PA resources for SOT^g^ population. Facilitators of PA included desire to live healthy lives and honor the donors, renewed physical abilities, and access to appropriate fitness guidelines and facilities. Desired features included safe PA guidelines, a reward system, affordability, integration of multiple functions specifically designed for after SOT, and the ability to share information with HCPs and peer recipients.
Vanhoof et al [36]	IHT^h^	Use of ICTs^i^ Willingness to use IHT as self-management support Technology acceptance Preferences for specific IHT features: 35-item newly designed interview questionnaire	Only 27.9% of participants had a smartphone, whereas 72.1% owned a computer with internet access. Participants gave a median score of 7 on a 10-point scale on whether they thought IHT was important to support their self-management. Technology acceptance: Patients who were single, married, or living together>patients who were widowed or divorced; Patients with high school education or lower>patients with college education or higher; Patients with previous technology use>patients without previous technology use Patients preferred automatic data transfer over sending data by themselves and feedback via visual aids over SMS text messages.
Wedd et al [37]	Patient portal (internet)	Use of web portal (whether a patient had any recorded activity based on Cerner server logs at any point during the study period)	48.2% of LTR participants used the patient portal LTR portal use: White>Black (*P*=.003); college education or higher>high school education or lower (*P*<.001) The patient portal was most frequently used for viewing laboratory test results by KTRs and LTRs (43.9% and 37%, respectively).

^a^LT: liver transplant.

^b^HCP: health care provider.

^c^eHEALS: eHealth Literacy Scale.

^d^KTR: kidney transplant recipient.

^e^LTR: liver transplant recipient.

^f^PA: physical activity.

^g^SOT: solid organ transplant.

^h^IHT: interactive health technology.

^i^ICT: information and communications technology.

**Table 3:** Characteristics of the intervention studies.

Study	Year	Country	Study design	Sample size, n	Age (y)	Sex (male; %)	Time since transplant
Barnett et al [38]	2021	Australia	Qualitative evaluation of a feasibility study	19	Mean 52	63	Median 4.4 years
Hickman et al [39]	2021	Australia	RCT^a^ with delayed intervention control	IG^b^: 23; CG^c^: 12	IG: mean 51; CG: mean 50	IG: 65; CG: 83	Median 4 years (IG) and 3 years (CG)
Ertel et al [40]	2016	United States	Prospective observational study	IG: 20; CG: 20	Mean 56	80	Not reported
Koc et al [41]	2022	Belgium	Prospective cohort study	Autonomous IG: 39; nonautonomous IG: 48; CG: 28	Median 59.1 (autonomous IG); 67.2 (nonautonomous IG); 66.0 (CG)	61.5 (autonomous IG); 60.4 (nonautonomous IG); 50 (CG)	Median 6.4 years (autonomous IG); 7.4 (nonautonomous IG); 7.0 (CG)
Lee et al [42]	2019	United States	RCT	IG: 50; CG: 50	IG: median 60; CG: median 58.5	IG: 52; CG: 60	Not reported
Tian et al [43]	2021	China	RCT	IG: 52; CG: 50	Mean 46.65	70.6	Not reported
Andrä et al [44]	2022	Austria	Prospective cohort study	124 (IG: 42)	Mean 63.2 (IG: mean 55.4)	74.1	Mean 6.5 years
Melilli et al [45]	2021	Spain	Prospective observational trial	84 KTRs^d^ and 6 LTRs^e^	Mean 46	73	Mean 69 months
Serper et al [46]	2020	United States	RCT	61 KTRs and 66 LTRs	Mean 52	64	Median 9.5 months
Zanetti-Yabur et al [47]	2017	United States	Prospective cohort study	67 KTRs and 7 LTRs (IG: 21; CG: 53)	IG: mean 52.6; CG: mean 54.1	60.8	Not reported

^a^RCT: randomized controlled trial.

^b^IG: intervention group.

^c^CG: control group.

^d^KTR: kidney transplant recipient.

^e^LTR: liver transplant recipient.

**Table 4:** Results of the intervention studies.

Study	Intervention	Control	Measurement timeline	Variable (measure)	Outcome
Barnett et al [38]	Telehealth-delivered diet and exercise program	—^a^	After the intervention (at 12 weeks)	End-user experiences Perspectives Program feasibility: thematic analysis of semistructured interviews	Telehealth reduced the perceived pressure and burdens of face-to-face care. Due to employment, participants desired flexible access to the program. Mediterranean diet was well accepted through enhancing confidence, finding practical solutions, and integration into family meals. Through tailored exercise prescriptions, participants experienced increase in exercise confidence, awareness of their exercise capabilities, and the ability to self-direct and prioritize exercise routine.
Hickman et al [39]	Telehealth-delivered lifestyle intervention	Delayed intervention control (12-24 weeks)	At baseline After the intervention (at 12 weeks in IG^b^ and 24 weeks in CG^c^)	Feasibility: recruitment rate, attendance rate, attrition rate, self-reported adequacy, adverse events, and perception of safety Dietary adherence: MEDAS^d^ QOL^e^: SF-12^f^ Metabolic syndrome: MetSSS^g^	Recruitment rate: 26% Attendance rate: 60% (diet: 71%; exercise: 52%) Attrition rate: 22.9% Staff-reported adequacy: 90% of sessions Participant-reported confidence of ≥8/10: 96% of sessions Participant-reported adequacy: 91% of sessions Adverse events and perception of unsafeness: none Increase in MEDAS: IG>CG (*P*=.004) MCS-12^h^: IG>CG (*P*=.03); increase in IG after the intervention (*P*=.03) Decrease in MetSSS after the intervention from before the intervention in the IG (*P*=.01)
Ertel et al [40]	Educational video program and telehealth monitoring of vital statistics using Bluetooth peripheral	Standard care (historic control)	At 30 days At 90 days	30- and 90-day readmission rate Effectiveness: 9 binary questionnaires Satisfaction: six 5-point Likert-scale questionnaires	30-day readmission rate: 20% (IG) and 40% (CG) 90-day readmission rate: 30% (IG) and 45% (CG; statistical analysis not conducted due to small sample size) Report of video program being helpful: 100% (response rate: 95%) Mean composite satisfaction score: 29/30 (IG and CG)
Koc et al [41]	Telemedicine-based remote monitoring program	Standard follow-up	At baseline After the intervention (median follow-up 2.0 years in the autonomous IG, 2.1 years in the nonautonomous IG, and 2.4 years in the CG)	Feasibility: initiation and continuation rate of autonomous CDSS^i^, and satisfactory score Safety: outpatient visits, emergency visits, unanticipated hospitalizations, tacrolimus level determinations, tacrolimus concentration, liver graft rejections, and retransplantations	75.7% of eligible patients initiated the program. 44.8% in the IG continued to use the CDSS (autonomous IG group) Median satisfactory score: 4/5 in IG Number of outpatient visits at follow-up: IG<CG (*P*<.001) Number of hospitalizations, rejections, or retransplantations did not differ between groups. Tacrolimus level determinations: IG>CG (*P*<.001 and *P*=.003 for the autonomous and nonautonomous IG, respectively) Tacrolimus blood level concentrations could be maintained lower after intervention than before in the IG (*P*=.04 and *P*=.002 for the autonomous and nonautonomous IG, respectively).
Lee et al [42]	THMP^j^	Standard care	At discharge At 30 days At 90 days	90-day readmission rate QOL: SF-36^k^ THMP participation: frequency	90-day readmission rate: IG<CG (28% vs 58%; *P*=.004) SF-36—physical functioning increase in IG (*P*=.02); general health increase in IG (*P*=.05) Frequency of vital sign monitoring and use of the devices to input the data was 86%.
Tian et al [43]	Telemedicine-based follow-up management	Usual care	At baseline At 30 days At 12 months	Hospital use 30-day readmission rate Survival rate, mortality, and morbidity	Length and expense of initial hospitalization were lower in IG than CG (*P*=.03 and *P*=.049, respectively). 30-day readmission rate: IG<CG (*P*=.02) Cumulative survival rate: no substantial difference but a 2-year stable period after transplant in IG Survival rates at 12 months: 94.2% (IG) and 90% (CG; *P*=.65) Occurrence of complications: no significant difference
Andrä et al [44]	Medication tracking and healthy lifestyle management app	—	At baseline After the intervention (at 2 months)	Basic requirements and knowledge about the app Potential benefits and expectations regarding the app Usability of and satisfaction with the app	57.3% owned a mobile device. 14.5% had previously used a health care app and had a younger mean age than that of the overall cohort. 31.8% of those requiring medication reminders relied on their spouses and were older than those using electronic alarms. 86.3% expected to obtain helpful information from the app. The individuals who tried the app (33.8%; IG) had a younger mean age than that of the overall cohort. 47.6% of app users used all its functions. 66.7% found the app to be adequate. 57.1% used the app daily for 2 months. The most frequently used functions were information access, reminders, and medication management.
Melilli et al [45]	App to monitor immunosuppressant adherence, classified into regular users, random users, and nonusers	—	At baseline At 3 months At 6 months At 1 year	Engagement with the app: willingness to use and continuation rate Medication nonadherence: number of missing drug intakes or doses taken out of time and nonadherent profiles according to distinct clinical characteristics	68% were regular users up to 6 months after the intervention. Of the regular users, 59% remained active up to 1 year after the intervention. Correct dose intakes (regular users): 69%-76% Intakes out of time (regular users): 12%-19% Missed doses (regular users): 9%-12% 4% of patients with high out-of-time intakes were LTRs^l^. Intakes out of time of >20% was the only variable independently predicting a tacrolimus CV^m^ of >30%. No differences in adherence levels were observed in relation to the type of immunosuppression formulations or combinations as well as the main clinical and demographic patient characteristics.
Serper et al [46]	Home-based exercise program using wearable devices, health engagement questions, and loss-framed financial incentives	CG arm 1: standard instructions regarding healthy diet and physical activity; CG arm 2: accelerometer without financial incentives	At baseline Daily (steps) At 4 months	Weight change Proportion of participant days in which ≥7000 steps were achieved	At 3 months, there were no significant differences in weight change across the 3 arms. 62% of the IG achieved ≥7000 steps compared to 45% of the CG arm 2 (*P*<.001). LTRs were associated with lower likelihood of achieving ≥7000 steps (*P*=.001).
Zanetti-Yabur et al [47]	Mobile app with medication-taking alarm system	Non–app users	At 3 months	Medication beliefs: BMQ^n^ Adherence: MMAS-8^o^ Patients’ ability to recall immunosuppressive treatment: IAT^p^ Serum tacrolimus, creatinine, and rejection assessment	Participants had adverse views about medication. CG participants were more likely to have negative beliefs about medicine in general compared to IG participants (*P*=.006). No difference in adherence patterns between IG and CG IG generally scored higher than CG in IAT, but this was not statistically significant (*P*=.19). No difference in laboratory test results or rejection

^a^Not applicable.

^b^IG: intervention group.

^c^CG: control group.

^d^MEDAS: Mediterranean Diet Adherence Screener.

^e^QOL: quality of life.

^f^SF-12: 12-item Short Form Health Survey.

^g^MetSSS: Metabolic Syndrome Severity Score.

^h^MCS-12: Mental Component Summary–12 (vitality, social functioning, role limitations due to emotional health, and mental health).

^i^CDSS: clinical decision support system.

^j^THMP: telemedicine-based home management program.

^k^SF-36: 36-item Short Form Health Survey.

^l^LTR: liver transplant recipient.

^m^CV: intrapatient variability.

^n^BMQ: Beliefs About Medicines Questionnaire.

^o^MMAS-8: Morisky Medication Adherence Scale-8.

^p^IAT: immunosuppression assessment test.

### Intervention Characteristics

Tables 5 and 6 summarize the details of the interventions reported in the selected studies. The most frequently addressed aspect of self-management was lifestyle education (6/10, 60%). Other topics of the interventions, allowing for overlap, included telemedicine-based remote monitoring (4/10, 40%) and medication management (3/10, 30%). The technological methods to deliver the interventions varied, including mobile apps (4/10, 40%; one of which included a wearable app), a 2-way videoconference portal (3/10, 30%), a web-based platform (1/10, 10%), Bluetooth peripherals (2/10, 20%), and a robot (1/10, 10%). The duration of the interventions ranged from short term (2 weeks after transplantation) to long term (up to 2 years after transplantation), with most (6/10, 60%) lasting 12 weeks.

Among the 6 studies that involved lifestyle interventions, 3 (50%) used synchronous video streaming to deliver exercise or diet educational sessions [38,39,43]. A total of 33% (2/6) of the studies offered mobile apps, with 17% (1/6) of the studies encompassing both lifestyle education and medication management [44]. The latter study provided information on self-management issues and allowed patients to self-document in a patient diary [44]. Another study provided a wearable accelerometer app, which was paired with financial incentives and questions regarding health engagement available on a patient portal [46]. A total of 40% (4/10) of the studies used telehealth to remotely monitor recipients’ vital signs and blood glucose levels [40,42,43] and conduct postoperative management regarding medication, gastrointestinal function, wound care, and laboratory test results [41-43]. For remote monitoring via telehealth, daily vital signs were collected using Bluetooth devices [40,42] or a robot [43].

In terms of medication management, 30% (3/10) of the studies used mobile app interventions that emphasized scheduled immunosuppressant intake [44,45,47]. These interventions used methods such as QR codes, reminder systems, and access to various resources (eg, medication and dose converter, medication lists) to facilitate medication adherence. In addition, 10% (1/10) of the studies, which delivered a telemedicine-based remote program, also monitored laboratory test results using an alarm system of predefined thresholds [41].

**Table 5:** Key features of the interventions.

Type of technology and study			Focus of self-management		Collaboration		Personalization		Adherence
**Videoconference portal**									
	Barnett et al [38]	Lifestyle management (diet and exercise education)		2-way videoconference portal		Exercise sessions were tailored to individual needs, capabilities, and preference for supervision.		Median attendance: 10 sessions out of 14	
	Hickman et al [39]	Lifestyle management (diet and exercise education)		2-way videoconference portal		Participants received up to 3 SMS text messages between sessions based on preference. At the end of the exercise sessions, participants received advice with personal prescriptions		Attendance rate: 60% (diet: 71%; exercise: 52%)	
**Telemedicine (website** **or** **tablet** **or** **mobile app** **or** **robot)-based remote monitoring**									
	Ertel et al [40]	Lifestyle education (posttransplant management) and remote monitoring (vital signs)		Not described		Not described		78% attended a listing class with educational information given at that time. 95% watched all the videos. 9 patients responded to ≥80% of the daily assessments. 12 patients recorded ≥80% of the daily vital statistics.	
	Koc et al [41]	Remote monitoring (laboratory test result management)		Not described		Not described		Patients in the IG^a^ entered 1526 (90.9%) of the 1679 required data items. 55.2% of the IG were not willing to report data in the CDSS^b^ and switched to the nonautonomous group but still communicated with the nurse. None switched to standard follow-up.	
	Lee et al [42]	Remote monitoring (vital sign management, posttransplantation education, and communication)		Video communication and phone calls were available.		Not described		Vital sign monitoring and use of devices: 86% Response to SMS text messages: 60% (0-30 days) and 25% (31-90 days) Use of video messaging or FaceTime: 6% Use of phone calls for issues: 70% Educational video slides viewed: 75%	
	Tian et al [43]	Remote monitoring (vital sign and posttransplantation management and communication) and lifestyle management (exercise)		Synchronous and asynchronous communication was available via robot, which was controlled by specialists via computer, phone, or iPad.		Not described		Not reported	
**Mobile app**									
	Andrä et al [44]	Lifestyle management and medication management		No direct connection with physicians		Not described		47.6% of app users used all its functions. 11.9% did not use the app at all. 57.1% used the app daily for 2 months.	
	Melilli et al [45]	Medication management		Not described		Not described		68% were regular users up to 6 months after the intervention. Among regular users, 59% remained active up to 1 year after the intervention.	
	Serper et al [46]	Lifestyle management (exercise)		Questions and answers related to health engagement were exchanged through bidirectional SMS text messaging.		Biweekly walking goals were tailored to their baseline based on the mean steps.		Adherence to target step goals was 74% in the IG. 84% of health engagement questions were answered, and among those, 95% were answered correctly. 92.1% (117/127) of participants were retained in the study.	
	Zanetti-Yabur et al [45]	Medication management		Not described		Not described		Not reported	

^a^IG: intervention group.

^b^CDSS: clinical decision support system.

**Table 6:** Summary of the interventions.

Type of technology and study		Adverse events	Reasons for attrition	Duration	Provider	TIDieR^a^ checklist score (1-12)
**Videoconference portal**						
	Barnett et al [38]	Not reported	—^b^	12 weeks	Dietitians and exercise physiologists	10
	Hickman et al [39]	None	Withdrew, no reason given (n=2) Lost to follow-up (n=6) Withdrew in delayed intervention control (n=4)	12 weeks	Dietitian and exercise physiologist	10
**Telemedicine (website** **or** **tablet** **or** **mobile app** **or** **robot)-based remote monitoring**						
	Ertel et al [40]	Not reported	—	Perioperative period education until 90 days after discharge and telemonitoring after discharge	Unspecified health care providers	8
	Koc et al [41]	Not reported	—	Median follow-up 2.0 years in the autonomous IG^c^, 2.1 years in the nonautonomous IG, and 2.4 years in the CG^d^	Physicians and specialized nurses	9
	Lee et al [42]	Not reported	Withdrew due to recurrent readmissions or death within 90 days after transplant (IG: n=1; CG: n=1)	3 months (daily monitoring)	Nurse care coordinators and providers	10
	Tian et al [43]	Not reported	Death (IG: n=3; CG: n=5)	2 weeks	Transplant specialists	9
**Mobile app**						
	Andrä et al [44]	Not reported	—	2 months	Unspecified health care providers	7
	Melilli et al [45]	Not reported	Lost to follow-up (n=2) Withdrew, no reason given (n=1)	12 months	Physicians	9
	Serper et al [46]	None	Died before study completion for a reason unrelated to the study (n=1)	2-week run-in period and 16 weeks of intervention	Unspecified health care providers	9
	Zanetti-Yabur et al [47]	Not reported	—	3 months	Unspecified health care providers	6

^a^TIDieR: Template for Intervention Description and Replication.

^b^Not applicable.

^c^IG: intervention group.

^d^CG: control group.

### Intervention Reporting and Comprehensiveness

On the basis of the TIDieR guide, the included intervention studies’ scores ranged from 6 to 10 (Tables 5 and 6). Detailed scores for each criterion can be found in Multimedia Appendix 2 [38-47]. All studies described the content and procedure of interventions, the type of technology used, and the locations where they were implemented. Of the 10 studies, 7 (70%) reported the duration and doses of the interventions, and 6 (60%) included the providers of the interventions. Personalized interventions were delivered to participants in 30% (3/10) of the studies. A total of 30% (3/10) of the studies included strategies or assessments to improve intervention fidelity, whereas 70% (7/10) of the studies monitored the adherence and fidelity of the delivered interventions. No intervention study reported any unforeseen changes in the interventions during the study process.

Table 7 shows how the selected interventions addressed the 5 core self-management skills identified by Lorig and Holman [14]. Of the 10 interventions, 4 (40%) included training in all 5 self-management skills. The number of core skills included in other studies ranged from 1 to 3. Taking action was a component in all interventions (10/10, 100%). Partnering with health care providers was featured in 80% (8/10) of the interventions, whereas Problem-solving was included in 70% (7/10) of the interventions. Decision-making was part of 60% (6/10) of the interventions, and Using resources was the least included, addressed in 50% (5/10) of the interventions.

**Table 7:** Core self-management skills addressed in the selected intervention studies.

Study	Problem-solving	Decision-making	Using resources	Partnering with health care providers	Taking action
Barnett et al [38]	●^a^	●	●	●	●
Hickman et al [39]	●	●	●	●	●
Ertel et al [40]	●	●	●	●	●
Koc et al [41]	○^b^	○	○	●	●
Lee et al [42]	●	●	○	●	●
Tian et al [43]	●	●	○	●	●
Andrä et al [44]	◐^c^	◐	●	◐	●
Melilli et al [45]	●	○	○	●	●
Serper et al [46]	●	●	●	●	●
Zanetti-Yabur et al [47]	○	○	○	○	●

^a^Reported.

^b^Not reported.

^c^Unclear.

### Study Outcomes

The use of and preferences regarding eHealth technologies were the most commonly examined outcomes in the descriptive studies. Study results concerning the use of technologies and its correlation with educational level and age varied. In the qualitative study by Lieber et al [33], 90% of the 20-person LT recipient sample reported using smartphones. In contrast, in the study by Vanhoof et al [36], only 27.9% of 122 solid organ transplant recipients owned a smartphone despite >70% having access to a computer with internet connection.

Concerning educational characteristics, 40% (2/5) of the studies found that individuals with college-level education or higher demonstrated greater eHealth literacy and more frequent use of the web patient portal than those with a high school education or lower [34,37]. Conversely, the study by Vanhoof et al [36] indicated that the group with a college education and higher had lower technology acceptance than those with a high school education or lower. Regarding age, younger patients had higher eHealth literacy in one study [34]. Another study found that those with previous experience using health apps, as well as those who tried the new app, had a younger average age than that of the entire cohort [44]. However, age was not a substantial factor in the willingness to use health technologies or patient portals in another 40% (2/5) of the studies [36,37]. Previous or routine use of health technologies was associated with higher eHealth literacy [34] and greater technology acceptance [36].

Moreover, 40% (2/5) of the studies highlighted that patients currently used smartphones or patient portals for reminders to take medications or to view laboratory test results. However, their preferences for future eHealth technologies extended beyond these uses. They expressed interest in connecting through web-based platforms with peer recipients and HCPs and gaining access to additional supportive features that included educational resources, medication management tools, and reward systems that consider affordability [33,35].

The most frequently measured outcomes in the intervention studies were adherence to the intervention and feasibility. A total of 80% (8/10) of the studies reported intervention adherence using various methods, including session attendance, device use frequency, response rate, and monitoring rate [38-42,44-46]. In 62% (5/8) of these studies, the overall adherence rate was >70% despite variability in measures [32,40-42,46]. Feasibility was measured using various methods, including qualitative interviews; recruitment rates; attendance rates; initiation and continuation rates; and reported levels of adequacy, confidence, effectiveness, or satisfaction [38-41]. Although rates of recruitment, attendance, initiation, and continuation varied between studies, the interventions were generally well received. Participant-reported satisfactory scores were >80% in 20% (2/10) of the studies [40,41], and participant-reported adequacy, confidence, and effectiveness levels were >90% in 30% (3/10) of the studies [39-41]. In a qualitative study, participants expressed that perceived burdens of face-to-face care were reduced regarding travel, time, or pressure and that confidence level in exercise increased due to tailored and self-directed telehealth education sessions [38].

A total of 4 studies involving telemedicine-based remote monitoring included 30- or 90-day readmission rates (n=3, 75%) and QOL (n=2, 50%) as outcomes. Regarding readmission rates, all studies reported a decrease in readmission rates after the interventions, 20% (2/10) of which were RCTs that reported a significant reduction in readmission rates [40,42,43]. A total of 20% (2/10) of the studies reported improvement in varying components of QOL, such as mental and physical function and general health [39,42].

Due to the heterogeneity of the interventions, various clinical outcomes were measured, such as dietary or medication adherence, weight change, level of metabolic syndrome, and tacrolimus drug level. Among the studies that incorporated mobile app interventions for medication management, the study by Melilli et al [45] reported that correct dose intakes on schedule occurred 69% to 76% of the time among the regular users of the delivered mobile app, whereas the study by Zanetti-Yabur et al [47] reported no difference in medication adherence on a validated scale between the intervention and control groups. Positive results on dietary adherence and metabolic syndrome were reported on a telehealth-delivered diet and exercise education program [38,39], but there was no significant change in weight after a home-based exercise program using a wearable accelerometer [46]. In a study incorporating remote monitoring focused on laboratory test results, tacrolimus level determinations were higher and blood level concentrations remained lower in the intervention group [41]. Regarding the reporting of adverse events, most studies (8/10, 80%) did not provide specific descriptions. However, 20% (2/10) of the studies reported no adverse events during the intervention period [39,46].

## Discussion

### Principal Findings

In this scoping review, we investigated the breadth of application and associated factors of eHealth-based self-management strategies in adult LT recipients. While previous literature on self-management among LT recipients has primarily focused on medication adherence, alcohol abstinence, and health maintenance (eg, smoking cessation, vaccination, and health screening) [15], the eHealth-based self-management studies in our review predominantly covered lifestyle management, medication adherence, and remote monitoring. Given the heterogeneity of the interventions and study measures, we assessed outcomes by describing trends, consistencies, or discrepancies among studies reporting similar outcomes. Although significant effects on reducing readmission rates or improving QOL were observed, synthesizing quantitative outcomes was not feasible due to a small number of RCTs. However, based on outcomes measured in various ways and participants’ qualitative reports, the interventions were well received, with generally high levels of feasibility, adherence, and satisfaction. The lifestyle management interventions used various modes of delivery, such as videoconferencing, web-based prerecorded videos, mobile apps, and patient portals. Remote monitoring was facilitated through a range of telehealth platforms, whereas interventions for medication adherence mainly used mobile apps. Notably, our review found that none of the eHealth-based self-management studies addressed the topic of prevention and management of alcohol relapse, a well-known concern among LT recipients [48].

Compared to intervention studies focusing on lifestyle management, the intervention studies focusing on medication adherence included relatively basic features, such as alarm reminders and logs for tracking medication intake. While these features target the action of medication taking, they fall short in promoting other essential skills such as decision-making or effective resource use to improve adherence to immunosuppressants. Given the complexity and variety of self-management behaviors required for LT recipients, eHealth technologies should be designed to support them in navigating multiple concurrent problematic situations to integrate disease management into their daily lives [15]. This objective extends beyond ensuring compliance with self-management behaviors and requires strategies to improve the self-efficacy of LT recipients and foster self-tailoring strategies [14]. This involves integrating their values, preferences, and readiness to increase motivation and confidence while also considering the various challenging scenarios that these LT recipients face, such as managing multiple medications due to comorbidities, coping with side effects, and handling varying schedules [9]. In interventions focused on lifestyle management and remote monitoring, features such as prerecorded or synchronous education sessions and platforms for asking questions and receiving answers were available. Such components could be beneficial in improving immunosuppressant adherence. In addition, incorporating elements such as role-play and quizzes that reflect challenging medication-taking scenarios along with web-based chatbots developed using frequently asked question algorithms and supplemented with emergency hotlines could enhance the decision-making and resource engagement skills of LT recipients.

While most of the reviewed studies (12/15, 80%) referred to the potential of eHealth in enhancing collaborative and individualized health care, only 33% (5/15) of them featured 2-way communication, and just 20% (3/15) incorporated personalized prescriptions or tailored goal setting. Videoconferencing emerged as the most common method for 2-way communication and building personalized strategies. Regular web-based meetings with HCPs or coaches and facilitators during these sessions can be effective, offering benefits such as reinforcing socially desirable behaviors; increasing accountability through clear, reciprocal goals and expectations; and enhancing interpersonal connectedness through support and feedback [49]. In addition, collaborative goal-setting strategies tailored to individual needs have been reported as effective in posttransplant recovery by acknowledging the variability in posttransplant health level, strength, and capacity among LT recipients [50]. The interest of LT recipients in connecting with peer recipients web-based also indicates a need for incorporating peer support groups to foster higher motivation and interpersonal connectedness [33].

In contrast, the studies that scored the lowest on the core self-management skills included minimal human collaborative elements such as communication or education sessions with HCPs [41,45,47]. These interventions primarily leveraged eHealth for its advantages in reducing labor-intensive tasks, enabling immediate evaluations, and improving accessibility while reducing exposure to infection sources [24,51,52] but overlooked the value of human support. Previous findings have suggested that digital person-to-person components can significantly improve effectiveness and adherence in eHealth interventions [49,53]. The selected interventions with lower levels of guidance and support included phone calls or SMS text messaging. However, assessing the relationship between the level of human support and postintervention outcomes was not feasible due to the heterogeneity of intervention strategies and the lack of clear causality between specific strategies and outcomes. Although it has been demonstrated that eHealth interventions with feedback channels are generally more effective than those without [49], further research is warranted to understand how the directiveness, interactivity, and immediacy of feedback impact the effectiveness of these interventions for sustainable behavior change in LT recipients with complex self-management needs.

In exploring untapped benefits of eHealth among the reviewed studies, we suggest that future research focus on developing predictive models and tailored interventions based on patient-generated data. The potential for creating algorithms to identify behavior patterns could be promising for personalized management or decision support systems. For instance, algorithms analyzing medication-taking logs could proactively identify individuals at risk, enabling more intensive monitoring to prevent medication errors or nonadherence [9]. The capacity of eHealth to collect extensive patient information remotely and conveniently should be maximized to create personalized management plans. By leveraging advanced data analysis and machine learning techniques, coupled with the incorporation of the preferences, needs, and circumstances of LT recipients, there is potential for a more sophisticated, patient-centered design of self-management interventions.

Another critical consideration when developing and implementing eHealth interventions is the age of the LT recipient population. Notably, in 87% (13/15) of the reviewed studies, the average age among the LT recipients was >50 years. Older age has been identified as contributing to the digital divide [54,55], potentially affecting the ability and access of LT recipients to self-management support using eHealth technologies [56,57]. While the proportion of LT recipients aged ≥65 years has increased in the past decade [58], research on older LT recipients has primarily revolved around graft function and long-term survival [59,60]. This indicates a need for further research addressing self-management and QOL in this demographic group [59,61]. The digital literacy of older adults should be assessed as a potential influential factor in eHealth self-management intervention studies [62]. The study by Andrä et al [44] examined the variability in mobile device use and usability with age as a key factor in the discrepancy between patients interested in the mobile app and those who actually used it. The study recommended a longer trial period and repeated training for the older individuals [44]. Moreover, intervention designs should accommodate older users or those with limited digital literacy. This could involve simplifying interfaces, using intuitive features, and providing clear instructions or support to aid their understanding and use of technology.

Furthermore, there is a pressing need for more studies on eHealth use and self-management outcomes among diverse ethnic groups and regions worldwide. The LT recipients in our review were primarily from North America and Europe, which does not proportionately represent the increasing number of LTs in many Asian countries. While the reviewed descriptive studies did not cover a wide array of eHealth-related characteristics, future studies examining the relationship between the digital divide and social determinants—such as ethnicity, educational level, economic status, health care access, and community resources among LT recipients—should consider the variability across different countries and regions. Such research would more accurately reflect the current global situation of LT recipients and validate the effects of eHealth in this population.

### Limitations

This review has several limitations. First, as we searched for articles explicitly including terms related to self-management based on previous literature on self-management among LT recipients, it is possible that articles including nuanced aspects of self-management were excluded. Second, it should be considered that the samples of the studies in this review comprised relatively old individuals as our review specifically focused on adult LT recipients. Thus, caution should be exercised when interpreting findings as age may influence adherence and self-management outcomes. As adherence and self-management needs significantly differ across developmental stages, future reviews focusing on younger populations are warranted. Third, this review included only studies published in English. Therefore, we may have missed relevant studies published in non-English languages, which should be considered with our finding related to the disproportional geographical distribution of the studies. Finally, it was inherently challenging to stratify and compare results for a detailed synthesis because this scoping review involved a small number of studies with heterogeneous designs, aims, and contents. Consistent with the purpose of the scoping review approach [63], our focus was on providing an overall mapping of the identified literature in this topic area rather than conducting an in-depth comparison of quantitative findings. Because the topic of eHealth interventions for self-management after LT is in its infancy but rapidly evolving, analyzing the replicability of the interventions to date using the TIDieR checklist may provide better insights for researchers and clinicians interested in further advancing this topic area. We suggest that future reviews prioritize analyzing the effectiveness of eHealth-based self-management interventions on various health outcomes and examine the interactions of social determinant factors as more evidence becomes available.

### Conclusions

This scoping review has highlighted the significant potential and emerging challenges of eHealth-based self-management strategies for LT recipients. The reviewed studies predominantly focused on lifestyle management, medication adherence, and remote monitoring. However, there is a noticeable gap in eHealth research concerning alcohol recidivism and the psychosocial and cognitive dimensions of progressing and evaluating self-management (eg, self-efficacy and self-regulation). Future research should aim to develop tailored eHealth interventions that encompass multifaceted elements of self-management skills. These interventions should not only leverage the benefits of technology but also incorporate digital human-to-human interactions to adequately address the complex needs of LT recipients. In addition, ensuring inclusive and equitable self-management support requires addressing the challenges of digital literacy, catering to the unique needs of older LT recipients, and considering the sociocultural contexts of LT recipients from diverse geographic regions.

## Appendix

Multimedia Appendix 1 Summary of database search strategies.

Multimedia Appendix 2 Detailed scores according to the Template for Intervention Description and Replication checklist for the selected intervention studies.

## Abbreviations

HCP: health care provider
LT: liver transplantation
PRISMA: Preferred Reporting Items for Systematic Reviews and Meta-Analyses
QOL: quality of life
RCT: randomized controlled trial
TIDieR: Template for Intervention Description and Replication
