# Supplementary material for: Self-Management Using eHealth Technologies for Liver Transplant Recipients: Scoping Review
Source: J Med Internet Res. 2024 Jul 4;26:e56664. doi: 10.2196/56664 (PMC11258531; doi:10.2196/56664)
Supplement: Multimedia Appendix 1 [file jmir_v26i1e56664_app1.docx]

**Multimedia Appendix 1**

Search date: June 19, 2023

PubMed

| Search number | Query |  |
| --- | --- | --- |
| 5 | 1 AND 2 AND 3 AND 4 | 1,196 |
| 4 | (((Liver Transplantation[MeSH Terms]) OR ("liver transplant")) OR ("liver transplant recipient")) OR ("Hepatic Transplantation") | 72,152 |
| 3 | adult[MeSH Terms] | 7,934,544 |
| 2 | (((((((((self-management[MeSH Terms]) ) OR (self-care)) OR ("health behavior")) OR (nutrition)) OR (alcohol)) OR ("medication adherence")) OR (medication)) OR (exercise)) OR ("physical activity") | 9,791,930 |
| 1 | ((((((((((((((((telemedicine[MeSH Terms]) OR (ehealth)) OR ("digital health")) OR (telehealth)) OR ("internet of things")) OR (computer)) OR (mobile)) OR ("mobile health")) OR ("mobile phone")) OR ("smart phone")) OR (internet)) OR (application)) OR (textmessag*)) OR (wearable)) OR (video)) OR (telephone)) OR ("virtual reality") | 3,816,415 |

CINAHL

| Search number | Query |  |
| --- | --- | --- |
| 4 | 1 AND 2 AND 3 | 26 |
| 3 | ((((((((((((((((((telemedicine+) OR (ehealth)) OR ("digital health+")) OR (telehealth+)) OR ("internet of things")) OR (computer)) OR (mobile)) OR ("mobile health")) OR ("mobile phone")) OR ("smart phone")) OR (internet)) OR (application)) OR (textmessag*)) OR (wearable)) OR (video)) OR (telephone)) OR ("virtual reality") OR (audio)) OR ("Telecommunications"+) | 529,025 |
| 2 | ((((((((((((((((self-management) ) OR (self-care+)) OR ("health behavior+")) OR (nutrition+)) OR (alcohol)) OR ("medication adherence")) OR (medication)) OR (exercise)) OR ("physical activity")) OR ("Alcohol Drinking+")) OR ("Alcohol Rehabilitation Programs+")) OR ("Medication Management")) OR ("Medication Compliance")) OR ("Drug Administration")) OR ("Self Administration")) OR ("Physical Fitness+") ) | 645,208 |
| 1 | (((Liver Transplantation) OR ("liver transplant")) OR ("liver transplant recipient")) OR ("Hepatic Transplantation") | 10,895 |

Web of Science

| Search number | Query |  |
| --- | --- | --- |
| 5 | 1 AND 2 AND 3 AND 4 | 76 |
| 4 | adult | 1,903,672 |
| 3 | (((Liver Transplantation) OR ("liver transplant")) OR ("liver transplant recipient")) OR ("Hepatic Transplantation") | 154,107 |
| 2 | (((((((((self-management) ) OR (self-care)) OR ("health behavior")) OR (nutrition)) OR (alcohol)) OR ("medication adherence")) OR (medication)) OR (exercise)) OR ("physical activity") | 2,786,750 |
| 1 | ((((((((((((((((telemedicine) OR (ehealth)) OR ("digital health")) OR (telehealth)) OR ("internet of things")) OR (computer)) OR (mobile)) OR ("mobile health")) OR ("mobile phone")) OR ("smart phone")) OR (internet)) OR (application)) OR (textmessag*)) OR (wearable)) OR (video)) OR (telephone)) OR ("virtual reality") | 9,489,380 |

Embase

| Search number | Query |  |
| --- | --- | --- |
| 5 | 1 AND 2 AND 3 AND 4 | 134 |
| 4 | 'adult'/exp | 10,937,943 |
| 3 | 'liver'/exp AND 'transplantation'/exp OR 'liver transplant'/exp OR 'liver transplant recipient' OR 'hepatic transplantation'/exp | 160,129 |
| 2 | 'self management'/exp OR 'self care'/exp OR 'health behavior'/exp OR 'nutrition'/exp OR 'alcohol'/exp OR 'medication adherence'/exp OR 'medication'/exp OR 'exercise'/exp OR 'physical activity'/exp | 7,234,915 |
| 1 | 'telemedicine'/exp OR 'ehealth'/exp OR 'digital health'/exp OR 'telehealth'/exp OR 'internet of things'/exp OR 'computer'/exp OR mobile OR 'mobile health'/exp OR 'mobile phone'/exp OR 'smart phone'/exp OR 'internet'/exp OR 'application'/exp OR textmessag* OR wearable OR 'video'/exp OR 'telephone'/exp OR 'virtual reality'/exp | 750,431 |

PsycINFO

| Search number | Query |  |
| --- | --- | --- |
| 4 | 1 AND 2 AND 3 | 28 |
| 3 | (((Liver Transplantation) OR ("liver transplant")) OR ("liver transplant recipient")) OR ("Hepatic Transplantation") | 1,177 |
| 2 | (((((((((self-management) ) OR (self-care)) OR ("health behavior")) OR (nutrition)) OR (alcohol)) OR ("medication adherence")) OR (medication)) OR (exercise)) OR ("physical activity") | 518,734 |
| 1 | ((((((((((((((((telemedicine) OR (ehealth)) OR ("digital health")) OR (telehealth)) OR ("internet of things")) OR (computer)) OR (mobile)) OR ("mobile health")) OR ("mobile phone")) OR ("smart phone")) OR (internet)) OR (application)) OR (textmessag*)) OR (wearable)) OR (video)) OR (telephone)) OR ("virtual reality") | 769,545 |
