# Supplementary material for: Self-Management Using eHealth Technologies for Liver Transplant Recipients: Scoping Review
Source: J Med Internet Res. 2024 Jul 4;26:e56664. doi: 10.2196/56664 (PMC11258531; doi:10.2196/56664)
Supplement: Multimedia Appendix 2 [file jmir_v26i1e56664_app2.docx]

| **Multimedia Appendix 2. Detailed Score according to the TIDieR checklist for the Selected Intervention Studies** | | | | | | | | | | | | | |  |
| --- | --- | --- | --- | --- | --- | --- | --- | --- | --- | --- | --- | --- | --- | --- |
| **Study** | **1. Name** | **2. Why** | **3. What - materials** | **4. What - procedures** | **5. Who provided** | **6. How** | **7. Where** | **8. When and how much** | **9. Tailoring** | **10. Modifications** | **11. How well- strategies to improve or maintain intervention fidelity and adherence** | **12. How well- extent of intervention fidelity and adherence** | **TIDieR score** |  |
| Barnett et al [38] | ● | ● | ● | ● | ● | ● | ● | ● | ● | ○ | ○ | ● | 10 |  |
| Hickman et al [39] | ● | ● | ● | ● | ● | ● | ● | ● | ● | ○ | ○ | ● | 10 |  |
| Ertel et al [40] | ● | ● | ● | ● | ○ | ● | ● | ● | ○ | ○ | ○ | ● | 8 |  |
| Koc et al [41] | ● | ● | ● | ● | ● | ● | ● | ○ | ○ | ○ | ● | ● | 9 |  |
| Lee et al [42] | ● | ● | ● | ● | ● | ● | ● | ● | ○ | ○ | ● | ● | 10 |  |
| Tian et al [43] | ● | ● | ● | ● | ● | ● | ● | ● | ○ | ○ | ● | ○ | 9 |  |
| Andrä et al [44] | ● | ● | ● | ● | ○ | ● | ● | ○ | ○ | ○ | ○ | ● | 7 |  |
| Melilli et al [45] | ● | ● | ● | ● | ● | ● | ● | ● | ○ | ○ | ○ | ● | 9 |  |
| Serper et al [46] | ● | ● | ● | ● | ◐ | ● | ● | ● | ● | ○ | ○ | ● | 9 |  |
| Zanetti-Yabur et al [47] | ● | ● | ● | ● | ◐ | ● | ● | ○ | ○ | ○ | ○ | ○ | 6 |  |
| TIDieR: Template for Intervention Description and Replication  ●reported; ○ not reported; ◐unclear | | | | | | | | | | | | | | |
